# A review on auditory space adaptations to altered head-related cues

**DOI:** 10.3389/fnins.2014.00219

**Published:** 2014-07-25

**Authors:** Catarina Mendonça

**Affiliations:** Department of Signal Processing and Acoustics, School of Electrical Engineering, Aalto UniversityEspoo, Finland

**Keywords:** localization, recalibration, learning, training, generalization

## Abstract

In this article we present a review of current literature on adaptations to altered head-related auditory localization cues. Localization cues can be altered through ear blocks, ear molds, electronic hearing devices, and altered head-related transfer functions (HRTFs). Three main methods have been used to induce auditory space adaptation: sound exposure, training with feedback, and explicit training. Adaptations induced by training, rather than exposure, are consistently faster. Studies on localization with altered head-related cues have reported poor initial localization, but improved accuracy and discriminability with training. Also, studies that displaced the auditory space by altering cue values reported adaptations in perceived source position to compensate for such displacements. Auditory space adaptations can last for a few months even without further contact with the learned cues. In most studies, localization with the subject's own unaltered cues remained intact despite the adaptation to a second set of cues. Generalization is observed from trained to untrained sound source positions, but there is mixed evidence regarding cross-frequency generalization. Multiple brain areas might be involved in auditory space adaptation processes, but the auditory cortex (AC) may play a critical role. Auditory space plasticity may involve context-dependent cue reweighting.

## Overview

It is nowadays well accepted that there is great plasticity in the sensory systems. Sensory plasticity was once thought to be limited to early stages of life (Parks et al., 2004). However, it is now well established that it is a lifelong process (Gilbert et al., 2001), and plasticity in the auditory domain is no exception (Rauschecker, 1999). Analyzing how humans adapt to changes in auditory localization cues is an increasingly relevant topic. There are nowadays a growing number of technologies in the field of hearing that impact auditory space cues. Cochlear implants greatly disrupt cues (Rosen et al., 1999), since spectral information is displaced in the auditory nerve and binaural cues are changed. Adaptation processes are also observed in hearing loss (for a review see Keating and King, 2013), and may impact how subjects adapt to new hearing aids. Hearing aids themselves affect auditory cues and require substantial adaptation. But even normal listeners face the challenges of adapting to altered spatial cues, as more and more sound systems resort to sound spatialization technologies that replace individual cues.

Auditory localization cues are individualized, since they are mostly a product of the interaction between sound waves and the body, namely the head. When head features change, so do the localization cues. Auditory localization cues are classified as either binaural or monaural cues (Middlebrooks and Green, 1991; Blauert, 1997). Binaural cues are principally linked with localization in the horizontal plane (left-right), whereas monaural cues are more highly weighted in the vertical plane (top-down) and in front-back distinctions. Binaural cues are obtained by comparing the sound input to each ear. This input varies in frequency, but most importantly in time of arrival and level. Differences in time of arrival at each ear are called interaural time differences (ITD) and differences in level are called interaural level differences (ILD). Monaural cues are those cues that could be obtained by a single ear. They consist of the level at each frequency, and are frequently called spectral cues. All these elements have been manipulated, often together, in studies on adaptation to altered head-related auditory space cues. The purpose of this review is to provide an overview on such studies.

Articles in this field have analyzed such processes using different nomenclature. Here we refer to auditory space as the localization of auditory events, therefore this concept refers to the relation between an auditory scene and how it is perceived in the space domain. The concepts of learning, adaptation and recalibration have been used almost interchangeably in this field. Learning can be described as a more explicit change, the subject can be aware of the adaptation process. Adaptation can be described as any change, resulting from accommodation to altered cues. Auditory space recalibration implies that the change is not only local, but a general adaptation in the direction of restoring the perceptual accuracy. In this paper we most often use the concept of adaptation.

The scope of this review has been limited to adaptation processes due to changes in head-related cues. We made this selection due to the fact that there is limited evidence that humans improve localization accuracy when trained in normal, unaltered cues. Some studies report a modest improvement, while other show no effects (for a review see Wright and Zhang, 2006). Due to addressing only studies using altered head-related cues, several relevant studies using altered environment or altered audiovisual correspondences are not reported. There is also a focus on normal-listeners, since most studies focus on this population. However, most of the data reported here applies to impaired listeners and many studies simulate hearing loss adaptation processes. Finally, we put great emphasis on studies with human subjects, but we also approach animal studies, namely when analyzing the neurophysiological correlates of adaptation to altered sound localization cues.

The studies reported here have been conducted over decades, and range considerably in methods and nomenclature. This is partly due to the fact that there are contributions from fields as different as medicine, biology, psychology, and engineering. This review attempts to organize and uniformize concepts regarding methods and results. Finally, an overview is presented over proposed and potential explanations of the underlying adaptation processes. Data are presented according to the following structure: overview of methods to induce adaptation; general adaptation results; adaptation aftereffects; neurophysiological correlates; underlying processes; and concluding remarks.

## Methods to induce auditory space adaptation

### Nature of localization cue manipulation

A way of testing the human adaptations to altered head-related localization cues is to artificially produce a change to such cues. Here we present an overview of methods used to produce such changes. Clinical studies and methods that have never shown potential to induce adaptation have been left out. One such example is ear swapping (Young, 1928; Hofman et al., 2002). Presenting subjects with switched binaural input has been implemented for periods as long as 30 weeks, but adaptation has never been found.

#### Ear blocks

The most common method for auditory cue manipulation in human studies has been the use of unilateral blocks, in which one ear is plugged with a sound attenuating earplug. This method has also been used to simulate conductive hearing loss and analyze adaptation effects. The main effect of the ear block is to produce a sound level attenuation, and therefore alter ILDs, but ITDs are also changed. However, the ear blocks do not affect exclusively binaural cues, since they can produce frequency dependent attenuation (Kumpik et al., 2010). This approach has been implemented in animals (King et al., 2001; Kacelnik et al., 2006) and in humans. In humans, it can be placed intermittently or in long-term. When long-term blocks are applied, subjects return to their daily activities and receive consistent natural feedback from the cue perturbation during a given period of time (Bauer et al., 1966; Florentine, 1976; McPartland et al., 1997). When intermittent, blocks are applied only during the test sessions, and removed between sessions (Musicant and Butler, 1980; Butler, 1987 Strelnikov et al., 2011).

#### Ear molds

In this method, wearable molds are fitted to the subjects' pinnae, to induce anatomical changes to the outer ear. Sound frequency levels (spectral cues) are therefore altered for each source position. There have been three studies resorting to this technique in normal-hearing humans. In a study by Hofman et al. (1998), four subjects wore molds in both ears for a period of up to 6 weeks. These molds disturbed the direction-depending spectral shaping of the outer ear without producing sound attenuation. In another study, van Wanrooij and van Opstal (2005) applied a similar mold but only to one of the ears, thus creating only a partial spectral perturbation. Carlile et al. (2013) applied small ear molds to both ears, filling 40 percent of the outer ear. Subjects wore them for 10 days, during all waking hours.

#### Electronic hearing devices

Hearing devices, like hearing aids, containing an external microphone and an internal speaker, have also been used to alter the head-related auditory localization cues. This method is technically more demanding, but allows more manipulations and greater control over the cue changes. Two studies have implemented this technique on normal hearing humans. Javer and Schwarz (1995) introduced a constant time delay to one ear, producing an azimuth shift of 66° to the sound image. Held (1955) used two matched hearing devices and displaced the microphones by 20° in azimuth.

#### Altered head-related transfer functions

Sound localization cues are produced by one's own body and its interactions with sound waves. It is possible to synthesize sounds that include such cues through the use of head-related transfer functions (HRTFs). These functions consist of the impulse response and its Fourier transform between a sound source position and a listener's ear canal entrance (Wightman and Kistler, 1988; Gardner and Martin, 1994). The stimuli are most often synthesized by convolving the sound of interest with the impulse response corresponding to the desired sound source position. Because subjects vary greatly in their anatomy, so do the HRTFs. Therefore, for good localization, it is necessary to use individualized HRTFs. On the other hand, the use of non-individualized or altered HRTFs poses an opportunity to learn how humans can adapt and learn to localize with someone else's localization cues.

Shinn-Cunningham et al. (1998a,b) altered the HRTFs such that they displaced the filters laterally, away from the center, thus creating “supernormal” cues for frontal source discrimination. Zahorik et al. (2006) used a head-mounted display to present subjects with an audiovisual virtual world, rendered in real-time though head-tracking and using non-individualized HRTF-based sounds. Mendonça et al. (2012, 2013) trained subjects to localize with non-individualized HRTFs, analyzing generalization patterns and long-term effects. Parseihian and Katz (2012) compared adaptation to individual HRTFs, with adaptation to more or less altered HRTFs. By controlling the amount of change of the localization cues, they could analyze its impact on adaptation processes. Majdak et al. (2013) trained subjects in localizing HRTF-based sounds that were either warped in frequency or band-limited.

#### Audiovisual discrepancy

Although it is not within the scope of this review, auditory space adaptation through displaced visual and auditory information should be mentioned. In such approach, there is no alteration of the head-related sound localization cues. Instead, visual spatial information is shifted in order to become misaligned with auditory spatial information. Many studies have looked into this cross-modal adaptation effect. In animals, it is common to apply a long-term prism that displaces the visual information by a few degree (for a review, see King, 2009). In humans, visual information has been shown to induce fast auditory localization shifts in an effect called the ventriloquism aftereffect (e.g., Recanzone, 1998; Lewald, 2002; Kopčo et al., 2009). In the ventriloquism effect, the perceived position of a sound is realigned with that of a visual source when both are presented concomitantly, but in different positions. In its aftereffect, a shift of perceived auditory position is still observed, even after the visual information is removed. This effect reveals the impressive dominance of vision in human space perception. However, visual information is not necessary for auditory adaptation and it can even be less efficient than other methods (Kacelnik et al., 2006; Strelnikov et al., 2011; Carlile and Blackman, 2014).

### Training paradigms

The methods used to induce adaptation to the altered head-related auditory localization cues are presented in this section. Methods were organized into three subgroups that vary mostly in intentionality. In sound exposure, subjects learn implicitly, without necessarily being aware of the adaptation process. In training with feedback, subjects are aware of the adaptation process and follow a specific training program. In active learning, subjects are actively and engaged in the learning task, and can learn implicitly or explicitly. Despite being presented separately, the methods are not mutually exclusive. There have been a few studies using several methods at once (see Section Adaptation by training).

#### Sound exposure

Training by sound exposure involves introducing a change to the head-related localization cues and allowing subjects to spontaneously adapt by listening to the altered sounds. Studies on humans and animals with congenital hearing impairment fit in this category. Also, in animal studies, chronic changes can be applied to the ears and then tested over time (e.g., King et al., 2001; Kacelnik et al., 2006). In this paradigm, subjects learn implicitly by continuous multisensory feedback. Since in this method subjects are allowed to move freely, there is continuous motor and visual feedback, allowing for rich feedback that replaces training. Some studies in this paradigm consist of a pre-test, exposure period, and a final post-test (e.g., Held, 1955). But most commonly, there are also regular tests during the exposure period, to analyze the adaptation pattern. Sound exposure paradigms can be separated into two different classes: long-term exposure and intermittent exposure.

In long-term exposure, experimenters apply the change to the localization cues, and then subjects use them continuously until the end of the experiment. Florentine (1976) had subjects wear a long-term unilateral block for a period of either 5, 22, 27, or 101 days for each of the four test subjects, respectively. van Wanrooij and van Opstal (2005) applied a long-term (9–49 days) monaural spectral perturbation. Held (1955) applied electronic hearing devices to his subjects for 8 h and allowed them to carry on with normal daily activities. In Hofman et al. (1998) subjects wore molds for up to 6 weeks and were tested regularly. Bauer et al. (1966) applied a long-term ear block for 65–67 h (Experiment 1) and had frequent tests to monitor evolution. Javer and Schwarz (1995) had their normal hearing subjects wear hearing aids during all waking hours for 3–5 days. McPartland et al. (1997) had the subjects wear an ear block over a period of 1week. They implemented tests before, during, and after the week of blocking. Carlile and Blackman (2014) applied binaural ear molds to subjects for a period of up to 60 days, until adaptation plateaued. Subjects wore the mold during all waking hours of the day. They were tested before the mold fitting, regularly during the adaptation period, and after mold removal.

In intermittent exposure, subjects only contact with the altered sound localization cues during the experimental sessions, and keep their natural hearing between sessions. Musicant and Butler (1980) blocked the right ear canal of eight participants, only during the test sessions. In one of their experiments, Shinn-Cunningham et al. (1998a,b) also exposed their subjects to altered sounds only during the experimental sessions.

#### Training with feedback

As in other paradigms, training with feedback most often includes a pre-test, the training process, and a post-test. The typical training process consists of sound localization tasks followed by a feedback, either classifying the response as right or wrong (response feedback) or specifying the true location of the stimulus (positional feedback).

In humans, response feedback is often presented in the form of a symbol or word. Butler (1987) trained subjects in an azimuth localization task. There was always response feedback, in the form of a word “correct” or “incorrect.” Irving and Moore (2011) implemented training sessions in which subjects had to localize sounds produced by an array of speakers. After response, there was feedback in the form of a green or red light, for *correct* or *incorrect* respectively. In training paradigms with positional feedback, after the subject points to the perceived auditory source position in space, the correct location is displayed. Bauer et al. (1966) had long-term unilateral plug combined with training (Experiment 2). Response feedback consisted of replaying the sound, combined with light flash, at the correct source location after the answer. Zahorik et al. (2006) trained subjects in sound localization and provided positional feedback by presenting, after each response, an audiovisual stimulus revealing the source position. Shinn-Cunningham et al. (1998a,b) presented positional feedback after each localization answer. This feedback consisted of a light flash at the correct location. Majdak et al. (2013) had an extensive feedback program. After response, a visual marker was displayed at the correct stimulus position. Subjects were required to find the source and point at it. Then, subjects returned to the original position, and the same sound was presented again, this time with the visual marker on. Subjects, again, had to find and point at the stimulus. Strelnikov et al. (2011) compared training methods. One group had only sound exposure; the second group had response feedback, with the presentation of the words “correct” and “incorrect” after response; and a third had positional feedback, with the presentation concomitant light and sound. Some researchers combined sound exposure, positional feedback and response feedback (Kumpik et al., 2010; Carlile et al., 2013). In these studies, the training sessions indicated not only the position of the stimulus, but also the magnitude of the response error.

#### Active learning

In this training type, subjects are actively engaged in the activity leading to auditory space adaptation. There are no predefined stimulus presentations or predetermined feedback. Stimulation is mostly a result of the subjects' own actions. However, unlike in sound exposure, there are specific sessions designed to accelerate the adaptation.

Parseihian and Katz (2012) used an implicit training method. The authors trained subjects in a virtual auditory environment. There was a game-like scenario in which subjects explored and localized auditory stimuli with a hand held tracked ball. While exploring, the subjects would hear an auditory virtual sound corresponding in space and time to the tracked ball. Though this perception-action coupling, the new HRTF-base sounds were learned.

Mendonça et al. (2012, 2013) used an explicit training method. They presented the subjects with an interface where they could select any of three to five source positions to be learned and play them freely. They were particularly encouraged to compare the differences between sounds. Subjects learned by actively studying the sounds. Play buttons were displayed in an array representing the source positions. Subjects had 5 min to complete the task. After the explicit learning phase, they went on to a phase of training with positional feedback, until all reached a criterion of 80 percent correct answers.

## Effects of auditory space adaptation

Effects found in auditory space adaptation studies are presented in two subsections, organized by training paradigms. The data presented focus on training length and adaptation found in studies with human subjects. Unfortunately, there is great variability across studies on the type of adaptation reported. Some studies reported results in terms of amount of stimulus needed to compensate for differences, some in terms of shift in auditory space (shifts in centroids), front-back confusions, polar/elevation/vertical angular error, lateral/azimuth/horizontal angle error, overall localization error, or even percentage of correct responses. Many studies also failed to provide clear numbers, reporting mostly statistical significances. As a consequence, comparisons across studies are somewhat difficult to achieve. Therefore, data are presented mostly regarding if adaptation effects were or not found, and what was the nature of such effects.

### Adaptation by sound exposure

Most studies using sound exposure used monaural blocks or ear molds to induce wearable cue changes. They then analyzed the evolution of subjects' localization abilities over time.

Adaptations in the horizontal plane have been reported in a number of studies altering mostly binaural cues, either by applying a unilateral block, or by changing binaural cues though a hearing aid. In a seminal work, Florentine (1976) applied a unilateral block to subjects. Subjects were tested daily for the first week and then every 48 h for the remaining time. There was also a pre-test and several post-tests upon plug removal. Test stimuli were pure tones a several frequencies. The adaptation period lasted for 27–101 days, but the author reported that, after 4–10 days of long-term use of a unilateral earplug (sound exposure), there was already a partial adaptation in centering auditory image. McPartland et al. (1997) had 6 subjects wear an ear block for 1 week. They tested their subjects with a pure tone localization task, during and after plugging. Four subjects revealed no change in lateralization during or after, while two subjects revealed effects during plugging. These results do not necessarily mean that subjects did not adapt. An alternative explanation would be that subjects adapted to every-day sounds, but could not extract horizontal localization cues from single frequency stimuli.

Held (1955) presented his subjects with sounds displaced in azimuth by 20° through an electronic hearing device and allowed them to experience these sounds freely for 8 h. To assess adaptation effects, the author tested subjects in an anechoic room prior to and after exposure. In the post-tests there was a displacement of auditory space halfway in the direction of the sound shift. Javer and Schwarz (1995) used binaural insert hearing aids to apply a constant time delay to one ear, thus altering the ITD. Subjects did not wear the device during the night. The shift produced after insertion was of approximately 66°. Tests were conducted in an anechoic chamber, where subjects had to localize sounds without feedback. Tests took place before device insertion and then at several intervals. Within hours of exposure, the displacement was reduced. The localization went on normalizing in subsequent days, but was never fully complete. Slattery and Middlebrooks (1994) used normal listening subjects and patients with congenital unilateral deafness. They applied a monaural plug to a group of normal listeners for a period of 24 h. The plug caused a prominent lateral displacement by an average of 30.9° toward the side of the open ear. Conversely, the patients had a considerable ability to localize, except for two patients that had a pattern very similar to the plugged group. After the 24-h period there was a slight trend toward improvement, with a reduction of 4.53° in lateral error, but there was great inter-subject variability, and therefore these differences were not significant. We hypothesize that this result may be due to the short exposure period used, having in mind that no specific training was used and that sound exposure studies usually last longer.

Musicant and Butler (1980) used intermittent exposure, by blocking the ear canal of participants in short localization sessions. They exposed the subjects only during testing sessions, and without any feedback. In a first test, they were exposed to 60 trials of broadband train bursts in a sound localization task. Then subjects performed only one trial per day, in a total of 60 trials. Finally, there was a post-test, similar to the first test. A second group skipped the pre-test. They showed that those subjects that had the first test had significantly lower errors in the 60 single trial sessions, than those without the first test. They also showed that, even without feedback, adaptation is possible, if enough exposure is provided.

Studies that analyzed adaptation in the vertical plane induced more prominent changes to the spectral localization cues, namely through the fitting of ear molds. Hofman et al. (1998) fitted molds to both ears of four subjects. Subjects were tested in elevation localization prior to fitting, and then regularly until plug removal. After mold insertion, localization in elevation was immediately impaired. During 23–39 days, subjects wore the plug at all time. Elevation localization was steadily reacquired throughout the experiment. van Wanrooij and van Opstal (2005) applied spectral perturbation only to one ear, by fitting an ear mold, and analyzed adaptations during a period of 9–49 days in elevation localization. Seven out of twelve subjects regained accuracy in elevation. The remaining five listeners varied in performance recovery. Subjects that were less perturbed in auditory cues were the ones that revealed less adaptation. Carlile and Blackman (2014) looked into adaptations inside and outside the visual field. They applied ear molds for 28–62 days (average 40.5 days), during all waking hours. Subjects completed two blocks of localization test at least twice a week, until performance gains plateaued. Subjects were also tested before insertion, immediately after, immediately before removal, after removal, and with the mold again 1 week after removal. Results were reported mostly in terms of front-back confusions. Front-back confusions were elevated immediately after mold insertion, but were gradually reduced during the adaptation period. Immediately after mold removal localization performance was found to overlap the baseline performance measured immediately before insertion. The patterns of adaptation were very similar both within and outside the visual field, showing that auditory space adaptations are not dependent on visual cues.

In sum, exposure to altered head-related localization cues seems to lead to gradual adaptations of auditory space. Time, stimuli and degree of cue change seem to affect the adaptation patterns.

### Adaptation by training

Bauer et al. (1966) were among the first authors to implement a specific training paradigm to induce adaptation in auditory space. With continuous usage of a monaural earplug (sound exposure), they obtained stabilization of localization improvement after 65–67 h. But when they added training with positional feedback, they found that improvement stabilization was obtained much faster, 5–8 h after start. Butler (1987) plugged subjects in one ear and administered training in five sessions, over a period of 2 weeks. He provided training with response feedback for sound sources varying in azimuth around the midline. After training, the displacement induced by the block was reduced.

Several authors implementing auditory space training programs compared feedback types. In Shinn-Cunningham et al. (1998a,b), subjects trained in with “supernormal” HRTFs gradually increased their lateralization resolution. Different experiments were conducted and there were two training paradigms. Half the subjects had training with positional feedback, while the others had speeded exposure to audiovisual pairs (positional feedback). Nevertheless, both groups showed a gradual adaptation to the altered cues. Strelnikov et al. (2011) applied intermittent unilateral ear blocks and trained subjects over five days, in one training session per day. There were three training groups: one with only sound exposure; one with positional feedback, where light and sound were presented simultaneously; and one with response feedback where after response subjects were told if response was correct or incorrect. Feedback was provided in only half of the trials. They found that improvement in azimuth localization upon plugging was obtained in both feedback conditions, but not in the sound exposure condition. Improvement was best for the group with positional feedback. Improvement with positional feedback was observed for all spatial regions, while improvement with response feedback was mostly in peripheral visual regions. Carlile et al. (2013) applied binaural ear molds for 10 days and compared training methods. All subjects had long-term exposure to the altered cues, since they wore the molds during all waking hours for the whole adaptation period. Additionally, there were four training conditions: only sound exposure; positional feedback in the form of a light indicating the sound source; positional and response feedback in the form of light and also sounds pulsing at a rate proportional to response error, subjects were also allowed to move their heads and explore the response feedback; same as the previous, but within a lit room. Training sessions were administered for 1 h daily. After the adaptation period, localization improvements, in terms of front-back confusions and elevation accuracy, were found in all combined training groups, but not in the no feedback group. The groups trained with positional and response feedback had significantly better results than the group trained only with positional feedback. The results in the no feedback group may be due to the short adaptation period used, since most studies with sound exposure last approximately twice as long (see next Section, Training type and Length). Nevertheless, it is quite surprising that the group with visual positional training did not reveal better results.

Other than comparing feedback types, some studies have implemented mixed training approaches. When trained to localize with altered HRTFs having rich multisensory and positional feedback, subjects reduced their front-back localization reversals after only two 30 min training sessions (Zahorik et al., 2006). In that study, participants were stimulated through a head-mounted display, in a virtual reality environment rendered in real-time as a function of subjects' movement. Therefore, in addition to the training with positional feedback, there was motor, visual, and auditory feedback by sound exposure. Localization accuracy was initially poor with frequent front-back reversals for five of six subjects. There was a general benefit of the training sessions, although the benefit was only observed on the front-back dimension. The richness of this training program might have contributed to the observed effects in such small amount of time. Mendonça et al. (2012, 2013) used an active learning paradigm with non-individualized HRTF-based stimuli. During the training session, subjects had to learn only a small sample of 3–5 sounds. In that session, subjects received explicit training for a period of 5 min, followed by training with positional feedback. All subjects reached the criterion of 80 percent accuracy in localizing the four/five selected sounds in less than 20 min. After this training procedure with selected sounds, there was an overall reduction of localization error in all other tested source positions, both in azimuth and elevation.

Irving and Moore (2011) also had a mixed training approach, combining both sound exposure and training with feedback. The authors compared participants with unilateral plugs and unplugged participants. All subjects had training prior to plugging (c.f. Section Adaptations to task, procedure, or auditory space?). Subjects that were plugged wore the plugs for 5 days. There were daily training sessions, lasting 45–60 min each, with response feedback. Stimuli were broadband noise bursts presented in the horizontal plane, 360° around the subjects. Despite the initial training, there was a large degree of inter-listener variability. Unplugged subjects improved slightly but significantly in localization until the last session. For subjects trained with unilateral earplugs, there was a steady growth of accuracy after the initial impairment.

Many questions remain open regarding the implementation of specific training procedures for auditory space adaptation. The type of feedback is only one of the many parameters that should be analyzed. The timing and duration of the training sessions, the selection of stimuli to use, and their relation to the degree to which auditory space cues have been changed are relevant questions that remain largely unexplored.

Kumpik et al. (2010) compared the timing and amount of training. Subjects were trained in localizing with a monaural earplug. Training consisted in positional and response feedback. One group did all training in 1 day, another did the same amount of training over a period of seven to 8 days, while a third group trained a larger number of trials over 8 days. Some subjects were trained in localizing sounds with constant flat spectrum, while others were trained in sounds with varying spectrum. Only subjects that were trained over 1week with predictable spectrum sounds showed adaptation by reducing the number of incorrect responses. This study revealed the benefit of spreading training in time, other than concentrating all training in a long session. Also, authors concluded that reliable spectral cues are needed for auditory space plasticity.

Parseihian and Katz (2012) compared directly the adaptation to different levels of head-related cue change. They trained and tested their subjects with HRTF-based sounds. The training task was a game-like scenario where blindfolded subjects could move freely. The interaction with the virtual world was through a hand-held position-tracked ball and sounds were spatialized at the hand position. Half subjects did all the training in 1day, while the other half had training sessions distributed over 3 consecutive days. There were three groups: one that trained in localizing with their own HRTFs; another that trained in localizing with non-individualized HRTFs that were close to their own, and another trained in very different HRTFs. Training sessions were three blocks of 12 min each. After the training sessions, the localization tests took place. No feedback was provided at that stage. Authors found that the group using individualized auralization started with, and kept, better localization results than the remaining groups. The greatest gain in performance was found after the first training session. Groups with only one training session had no significant improvement, but groups trained in 3 days did. Most of the improvement was found in decrease of vertical error. A slight improvement was found in horizontal error, in groups with good HRTFs (close to their own), but not bad. This revealed that possibly adaptation processes take longer when greater changes are applied.

Majdak et al. (2013) also compared different levels of cue change. They used a spherical virtual audiovisual environment with HRTF-based sounds. Subjects were trained with visual feedback 2 h per day, for 21 days. Prior to and after training, subjects were tested in localization of sounds with the original individualized HRTFs, and with low-passed, frequency warped, and band-limited versions of those HRTFs. Then they were trained in either the warped sounds or the band-limited sounds. Training was effective for both groups. However, those subjects that trained with frequency warped sounds started with much higher errors and never localized as well as subjects trained in band-limited sounds. Even after training, localization was not as good as with the subjects' original HRTFs. Results pointed out that subjects can easily adapt to narrower stimulation bands, which can be observed in hearing loss. Distortion of the frequency cues impact more auditory localization and lead to potentially longer adaptation processes.

In sum, implementing specific training paradigms or combined approaches seems to be highly effective, and thus a promising approach to induce auditory recalibration. Methods vary greatly, and different feedback modalities lead to different adaptation processes. It seems that the success of the training program depends on the nature of the task and feedback provided. Active learning may be a promising way to enhance adaptation. Also, combining approaches and providing sensory-motor engagement may provide for better learning conditions. Greater cue changes seem to lead to longer adaptation periods. On the other hand, several training sessions may be preferable to the use of intensive one-day training sessions.

### Training type and length

In this section we take a closer look into the various training types and associated effectiveness in terms of adaptation time. Table 1 presents a summary of studies on adult humans with normal listening. All these studies applied a change in localization cues and analyzed adaptation effects. Overall, auditory adaptation studies in humans vary greatly in length, from 10–20 min (Mendonça et al., 2012, 2013), to 27–101 days (Florentine, 1976). To obtain an estimate of average training length per study type, we computed local averages for studies in which length was itself variable. Two studies were not considered, due to having very irregular training methods (Musicant and Butler, 1980; Irving and Moore, 2011). Only methods that produced effects were considered.

**Table 1:** **Summary of studies on auditory space adaptation with normal-hearing human listeners**.

**Source**	**Type of cue change**	**Auditory stimuli**	**Type of training**	**Duration of training**	**Adaptation effects**	**After effects**
Held, 1955	Long-term hearing aid	Bandpassed noise	Sound exposure	8 h	Partial	–
Bauer et al., 1966	Long-term monaural block	Broadband noise	Sound exposure	65–67 h	Yes	Back to pre-plug levels
			Exposure, training with positional feedback (V)	5–8 h	Yes	Back to pre-plug levels
Florentine, 1976	Long-term monaural block	Pure tones	Sound exposure	27–101 days	Yes	Adaptation 7–15 days after removal
Musicant and Butler, 1980	Inttermittent monaural block	Bandpassed noise	Exposure without feedback	1 h + 1 trial/day over 60 days	Yes	–
				1 trial/day over 60 days	No	–
Butler, 1987	Inttermittent monaural block	Bandpassed noise	Training with response feedback (R/W)	1 h*5 (2 weeks)	Yes	Adaptation 2–2.5 months after training
Slattery and Middlebrooks, 1994	Long-term monaural block	Broadband noise	Sound exposure	24 h	No	–
Javer and Schwarz, 1995	Long-term hearing device	Bandpassed and broadband noise	Sound exposure	3–5 days	Yes	–
McPartland et al., 1997	Long-term monaural block	Pure tones	Sound exposure	1 week	Partial	–
Hofman et al., 1998	Long-term binaural ear mold	Broadband noise	Sound exposure	23–39 days	Yes	Back to pre-plug levels
Shinn-Cunningham et al., 1998a,b	Inttermittent altered HRTFs	Click trains	Training with positional feedback (V) (AVM); sound exposure	2 h*8 (2–6 weeks)	Yes	–
van Wanrooij and van Opstal, 2005	Long-term monaural mold	Bandpassed and broadband noise	Sound exposure 9–49 days	Partial (elevation)	Back to pre-plug levels soon after	
Zahorik et al., 2006	Inttermittent altered HRTFs	Bandpassed noise	Training with positional feedback (AV)	1 h*2	Yes	Effects lated over 4 months
Kumpik et al., 2010	Intermittent monaural ear block	Broadband noise	Training with positional (V) and response feedback(R/W)	1 day	No	Back to pre-plug levels
				~1 h *7–8 days	Yes	
Strelnikov et al., 2011	Intermittent monaural ear block	Broadband noise	Sound exposure	1 h*5 days	No	–
			Training with positional feedback (AV)		Yes	
			Training response feedback (R/W)		Yes	
Irving and Moore, 2011	Long-term monaural block	Broadband noise	Sound exposure; Training with response feedback (R/W)	5 days exposure; 5 h training	Yes	Immediately back to pre-plug levels
Mendonça et al., 2012	Inttermittent altered HRTFs	Broadband noise	Sound exposure (static)	10 blocks (1 h)	No	–
			Explicit training; Training with positional feedback (V)	10–20 min	Yes	–
Parseihian and Katz, 2012	Intermittent altered HRTFs	Broadband noise	Implicit training (AM)	3*12 min	Yes	–
Majdak et al., 2013	Intermittent altered HRTFs	Broadband noise	Training with positional feedback (V)	2 h*21 days	Yes	Same results 1 day later
Carlile et al., 2013	Long-term binaural ear mold	Broadband noise	Sound exposure	10 days	No	
			Sound exposure; Training with positional feedback (V)	10 days, 10 h training	Yes	–
			Sound exposure; Training with positional (AVM) and response feedback (level)	10 days, 10 h training	Yes	
Mendonça et al., 2013	Inttermittent altered HRTFs	Broadband noise and speech	Explicit training, training with positional feedback (V)	10–20 min	Yes	Effects lasted over 1 month
Carlile and Blackman, 2014	Binaural ear mold	Broadband noise	Sound exposure	28–62 days (average 40.5 days)	Yes	Back to pre-mold levels upon removal; adaptation still one

*Acronyms stand for sensory modality of feedback: V, Visual; AV, Audiovisual; AM, Audiomotor; AVM, Audiovisual motor; R/W, Right/Wrong; Level, Level of response error*.

We calculated that sound exposure studies lasted on average 20 days (*SD* = 22.4 days). Studies using training with feedback (both types) lasted an average of 18.8 h, (*SD* = 14.9 h). The active learning studies lasted an average of 22 min (*SD* = 12.12 min). Comparing training with positional feedback and training with response feedback, we find that training with positional feedback studies had longer adaptation periods: average 13.8 h against 5 h in studies with response feedback. Regarding two studies that mixed training with feedback and simple exposure (Kumpik et al., 2010; Irving and Moore, 2011; Carlile et al., 2013) the average training duration was 7.5 h (*SD* = 2.5 h).

There is therefore a clear benefit of training with feedback, comparing with sound exposure, and of active learning comparing to any other method. However, the number of studies using active learning is still too small to draw significant conclusions. Similarly, adaptation times reveal that response feedback can be associated to faster training processes than positional feedback, but differences are small and there are not enough studies to draw such comparisons in a conclusive way. Studies using training with positional feedback resorted mostly to visual, audiovisual and audiovisual motor information as feedback, while response feedback studies used words, colors, or pulsed sounds. It would be expected that the former feedback types, richer in spatial information, could lead to better adaptations. On the other hand, more symbolic feedback types may require the recruitment of additional attentional and memory resources, which are crucial for learning. Further studies are required to draw clear conclusions on the most efficient stimuli and methods to induce adaptations.

### Adaptations to task, procedure, or auditory space?

It may be argued that improvements in localization accuracy observed in these studies are the result of a task, or procedure learning, instead of actual auditory space adaptations. There isn't enough research to understand and predict how much of the adaptation effects can be accounted by task or procedure learning. Here we refer to task learning as a process in which the subject becomes acquainted with the stimuli and the judgment type, a cognitive adaptation which includes establishing an internal criterion for the decision on the stimuli. By procedure learning we mean the adaptation to the interface and response type. It comprises optimizing the process of perceiving-deciding-responding. Unfortunately, these processes are very task- and subject-specific, so there is no good rule to predict the extent of their effects or how long they take. Psychophysical studies with human subjects usually include a very short practice block before starting the data collection. This has been used in some auditory space learning experiments (e.g., Strelnikov et al., 2011). Alternatively, some authors have resorted to longer preliminary training blocks with the subject's unaltered cues.

Slattery and Middlebrooks (1994) provided all subjects with two brief training sessions to familiarize them with the testing procedure, before applying monaural plugs. In the first training session, a light would turn on over the speaker that displayed the sound. This procedure was repeated 15 times. In the second session the loudspeaker light only appeared after the subjects answered by localizing the sound. Kumpik et al. (2010) trained subjects in localizing broadband noise stimuli prior to applying monaural earplugs. Subjects were trained until they reached 85 percent correct answers, which could take up to 6 days. Interestingly, only the group trained in localizing these same sounds after plugging revealed significant adaptations, but the authors never debated the potential role of the preliminary training on the final results. Irving and Moore (2011) had one of the longest, most comprehensive preliminary training programs. They started by training participants with unaltered cues for 4 days. Then, half participants wore a plug for 5 days, while others did not. Carlile et al. (2013) and Carlile and Blackman (2014) trained their subjects in the testing procedure before applying changes to the ears. The procedure included pointing with the nose at the perceived sound source after stimulus offset. After the answer, a light was presented over the loudspeaker that presented the stimulus; then, noise bursts were displayed from that speaker at a rate inversely proportional to the pointing error. Subjects were therefore trained in pointing to the perceived position with greater accuracy. There were 150–200 training trials altogether.

A few concerns can be raised in an approach in which subjects are first trained on their own cues and only then on the altered cues. Auditory training may have unknown effects. It can increase plasticity and improve the success of subsequent adaptation processes (Linkenhoker and Knudsen, 2002). Alternatively, it can increase strangeness when cue alteration is first applied, artificially raising the initial error levels. Indeed, if subjects have just been trained in a task with specific cues, they may exhibit initial enhanced errors solely due to expectations of particular cue arrangements. Unfortunately, no studies exist comparing pre-test adaptation procedures.

In an ingenious approach, Majdak et al. (2013) trained subjects in the procedure, without affecting the baseline sound localization results. They had a preliminary training session, where subjects learned to identify the visual target and point at it. No sounds were presented during this session. This way, subjects became acquainted with the task, interface, and improved response precision, presumably without affecting the performance on the auditory task. One common way to separate localization improvement from mere task training effects is to have different tasks and setups for the training and testing sessions (e.g., Mendonça et al., 2013). In such cases, much like in the training by sound exposure, the improvement in the localization task cannot be attributed to successive training. However, even in such cases, there is cumulative experience in the testing procedure itself. The only way to account for this effect is to have a control group (e.g., Irving and Moore, 2011) that undergoes the testing without the training. In this case, the differences between groups can clearly be attributed to adaptation to the new cues, rather than adaptation to task and procedure.

## Adaptation aftereffects

### Durability

To analyze the adaptation aftereffects in the time domain, we must look separately into studies that implemented long-term cue changes and intermittent changes. Studies that implemented long-term changes looked into hearing and localization upon the removal of such changes. There are conflicting results at this level.

Florentine (1976) reported a post-experimental effect of 7–15 days after removing the unilateral blocks. Subjects still required an imbalance of channel loudness to perceive the auditory image as centered. Since this was an exceptionally long study (27–101 days), we hypothesize that the long-term unilateral mold induced some hearing loss to the blocked ear. This is in line with other findings showing that temporary conductive hearing loss leads to a binaural hearing impairment that lasts beyond the duration of the impairment (for a review, see Moore et al., 2001). No other study implementing long-term monaural blocks obtained such an effect, but no other occluded the ear for such a long period. Irving and Moore (2011) observed that, upon removal of the block, subjects localized again exactly as they did before insertion. Bauer et al. (1966) also tested localization shift after plug removal. In one experiment subjects wore plugs for 65–67 h. In the other experiment, for 5–7 and had additional training with feedback. Post-plug shifts were modest or neglectable, compatible with assumption that subjects went back to their natural auditory map. Held (1955) reported that, upon removal of the hearing device, subjects were localizing like they did in the pre-test. Hofman et al. (1998) found that, after removal of binaural molds, localization in elevation was close to the original levels. In a similar way, van Wanrooij and van Opstal (2005), Kumpik et al. (2010), and Carlile and Blackman (2014) found that soon after restoring the subjects' ears localization abilities were at the same level as before the adaptation period. On the other hand, in Carlile and Blackman (2014), 1 week after removal, when the mold was reapplied, localization was similar to that obtained at the end of the adaptation period, demonstrating that the learned cue-to-space relationships were still available.

In experiments that applied only intermittent changes, similar enduring results were obtained. In Butler (1987), the subjects only wore the earplug during training sessions. They trained for five sessions, over a period of 2 weeks. Adaptation was retained for a period of 2–2.5 months. Zahorik et al. (2006) tested their subjects 4 days and 4 months after training. Benefits in localization accuracy were still found 4 months later. Mendonça et al. (2013) tested their subjects 1 h, 1 day, 1week, and 1 month after training. Effects of training were still observed 1 month after training. Implications of these finding are discussed in Section Underlying Processes.

### Generalization

In perceptual learning, there are well known effects of specificity to trained attribute, position, orientation and context (Gilbert et al., 2001). Generalization occurs when the training-induced perceptual adaptation is found not only in the trained stimuli or task, but also in others. Generalization mechanisms are found in auditory learning, but they vary with task and stimuli, and are often limited to sound frequency (for a review, see Wright and Zhang, 2009). In auditory space learning with altered localization cues, findings are also often contradictory.

As already referred in Section Effects of Auditory Space Adaptation, Butler (1987) found that spatial adaptation was specific to trained cue spectrum, On the other hand, Zahorik et al. (2006) found that, after training, subjects improved in localizing not only the trained auditory source positions, but also other, untrained sources. A similar result was obtained by Mendonça et al. (2012). Mendonça et al. (2013) looked deeper into auditory space generalization patterns. They found that subjects trained in localizing sources varying exclusively in the vertical plane became better in localizing sources varying in the horizontal plane, and vice-versa. Subjects trained in localizing speech became better in localizing broadband noise, and vice versa. However, there was a benefit in training with broadband noise leading to improved learning and generalization levels. Finally, subjects were trained in only four stimuli positions, but revealed improvements in all subsequently tested positions. These results reveal the potential of using simplified training approaches to induce fast adaptations through generalization.

## Neurophysiological correlates

There is great plasticity in the neural circuits that process sensory information (Rauschecker, 1999). It is most relevant during infancy, as the body grows, but it is maintained in the adult brain (King et al., 2000, 2011). Learning produces changes in the brain, which can take the form of increases in dendritic length, spine density, synapse formation, increased glial activity, or altered metabolic activity (Kolb and Whishaw, 1998). It is natural to assume that auditory space adaptation processes take place in the auditory pathway, where space is processed. However, since there is no full understanding on how space is encoded at higher instances of the human brain, there are also many open questions on the substrates of the adaptation processes. Furthermore, there seems to be substantial difference among species on this matter.

The localization process starts with the extraction of direction-dependent cues in the brainstem (e.g., King et al., 2001), early in the auditory pathway. Interestingly, the olivocochlear system, involved in the descending control of the cochlea, has been shown to be unnecessary for accurate auditory localization, but it is involved in relearning auditory space during unilateral conductive hearing loss (Irving et al., 2011). ITD and ILD are predominantly, but not only, processed in the medial superior olive and lateral superior olive respectively (Moore et al., 2010) and these nuclei project to the central nucleus of the inferior colliculus (IC). The IC also receives input from the contra-lateral dorsal cochlear nucleus, where monaural spectral cues seem to be processed (Yu and Young, 1997; Zatorre et al., 2002). There are multiple feedback loops between the auditory cortex (AC) and IC (Huffman and Henson, 1990; Oliver, 2005), and therefore the IC also receives massive descending projection from the AC (Maeder et al., 2001).

### Inferior and superior colliculi

The IC is a midbrain nucleus of the ascending pathway (Maeder et al., 2001). It projects to the superior colliculus (SC) (Oliver and Huerta, 1992; King et al., 1998a). The SC has a topographical organization, where stimuli from different points in space activate different areas. It is mostly visual in the upper layer and multisensory in the lower layers (King and Palmer, 1983; Middlebrooks and Knudsen, 1984; King and Hutchings, 1987). So it has been proposed that there is a topographically aligned visual and auditory map in the SC (King and Palmer, 1983; Middlebrooks and Knudsen, 1984; King and Hutchings, 1987). This hypothesis has large support in several species, but remains open in primates.

Studies on animals raised with sensory impairment show that the map of auditory space in the SC is shaped during the development of both auditory and visual systems (King et al., 2000). In the barn owl, adaptation processes have been well documented. Plasticity at the level of the external nucleus of the IC is largely responsible for the frequency-dependent adjustments in ITD tuning that are observed in the optic tectum of owls raised with spectacles (Gold and Knudsen, 2000). The optic tectum is the homolog of the SC in the barn owl and contains mutually aligned neural maps of auditory and visual space (Brainard and Knudsen, 1993). There is a point-to-point projection from the optic tectum to the IC (Hyde and Knudsen, 2000). Therefore, this anatomical organization contributes to the visual calibration of the auditory space map at the IC (Brainard and Knudsen, 1993; Feldman and Knudsen, 1997; Hyde and Knudsen, 2000). In ferrets, the auditory spatial map is not as well organized, but activity in superficial layers of the SC is thought to play a role in the alignment of the topographical arrangement of the IC (King et al., 1996, 1998b). The SC in the mammal has many multisensory neurons in the deeper layers, thought to be responsible for a unified impression of the world, that activate selectively according to spatiotemporal constraints (Stein and Meredith, 1993). The upper layers of the SC are exclusively visual and are innervated by topographically organized projections from the retina and the visual cortex (Huerta and Harting, 1984). Therefore, despite a different organization at the IC, it is still reasonable to assume that the interactions between IC and SC might be related to auditory space adaptation in humans. This adaptation process would mostly be relative to visual and tactile spatial feedback.

### The cortex

In humans, both the AC and the posterior parietal cortex are involved in auditory localization (Griffiths et al., 1996; Zatorre et al., 2002). The parietal cortex is possibly involved in cross-correlating between auditory localization and head movement information (Rauschecker, 1999). The AC is key for auditory localization, since temporal lobe damage can lead to impaired auditory localization (Masterton, 1997; Clarke et al., 2000; King et al., 2011). The AC receives binaural inputs that are tuned to sound frequency, and it is organized in a tonotopic way. Preferred sound azimuths appear to be clustered across the AC (Imig et al., 1990; Rauschecker, 1999; Tian et al., 2001). The posterior AC responds to sounds that vary in spatial distribution, but only when multiple stimuli are presented together, implicating this cortical system in the disambiguation of overlapping auditory sources (Zatorre et al., 2002). It has been suggested that the sound localization mechanism based on spectral cues assumes a flat spectrum and compares the incoming sound with the acquired HRTF templates (Blauert, 1997). Therefore, regarding frequency-dependent cues, it would be more economical to adapt the spectral coding of sound localization by means of cortical plasticity (Rauschecker, 1999). Recent evidence revealed that the AC is involved in mammal auditory space adaptations. Nodal et al. (2012) reversibly deactivated different cortical areas of ferrets over a few weeks. The orientation of the animals to sounds was not affected by silencing any region of the AC, but the experience-dependent plasticity was. After plugging one ear, the localization recovery was not as complete in animals with the AC deactivated, compared to control animals. Also, selectively deactivating the cholinergic nucleus basalis that projects to the AC affects not only auditory localization, but also impairs experience-dependent auditory space adaptations (Leach et al., 2013). Additionally, the corticocollicular pathway, from the cortex to the IC, plays a crucial role in learning-induced auditory space plasticity of mammals. When these neurons were selectively killed in ferrets, the recovery in auditory localization after occlusion of one ear was impaired (Bajo et al., 2010). Keating et al. (2013) showed that the role of the AC in auditory space plasticity involves a reweighting of different spatial cues. Ferrets reared with unilateral plugs alternated with periods of normal hearing relied more on monaural cues than animals raised with normal hearing. This change in behavior was accompanied by changes in neuronal responses in the primary AC. However, this reweighting disappeared in periods of normal hearing, revealing that this type of adaptation is context-dependent.

## Underlying processes

In this section we attempt to bring together neurophysiological evidence and psychophysical data. We are still far from understanding the neuronal and cognitive mechanisms underlying auditory space adaptation processes. Keating and King (2013) proposed that adaptation may be achieved either by learning a new relationship between altered cues and points in space or by changing the way different cues are integrated in the brain. The former would consist of a cue remapping process, potentially involving structural changes in the brain, like the ones observed in the barn owl. The latter could involve a cue reweighting process. Cue processing would remain the same, but a different decision rule, regarding the corresponding position in space, would be applied. In a similar way, Shinn-Cunningham (2001) proposed that the auditory system is optimized for computing spatial location from normal spatial cues and short-term training cannot influence how spatial position is computed internally, but only how spatial percepts are associated to exocentric space. There is some evidence supporting the existence of cue reweighting processes in humans (Kumpik et al., 2010). This evidence pointed to the fact that, if such mechanisms were to exist, they should be context-dependent. In different contexts, different cue combination rules could be used.

As reported above, in most studies on the adaptation to a long-term cue change, such as fitting an earplug or mold, upon removal of the change subjects readily returned to localizing as they did before. An interpretation of these data is that subjects developed a new cue combination rule, between cues and perceived position. In these cases, the previous combination rule was not altered, and instead a new one was created for that new context. In a similar way, subjects that had intermittent training improved in localizing with the altered cues, while using their natural cue integration between experimental sessions. This continuous improvement of a second cue combination rule can only be explained if both rules were developed in parallel, and used one in each context.

van Wanrooij and van Opstal (2005) found that subjects that were less perturbed by a monaural ear mold improved less than subjects that had to deal with greater differences. The authors suggested that adaptation to spectral cue manipulations depends on the correlation between the new and the old cue combinations. In this case, before adaptation, there would be an analysis to correlate the perceived space and some form of feedback. If relevant differences were found, adaptation would take place.

All these findings point out that the brain might be able to develop and use different cue combination rules in parallel. This feature, despite computationally demanding, might be evolutionarily optimal. In real-world situations, not only the anatomy, but also the contexts, change considerably over time. The acoustic cues in a classroom or in a supermarket, for instance, are markedly different. But crucially, each context may remain stable over time (Keating and King, 2013).

**Figure 1:**
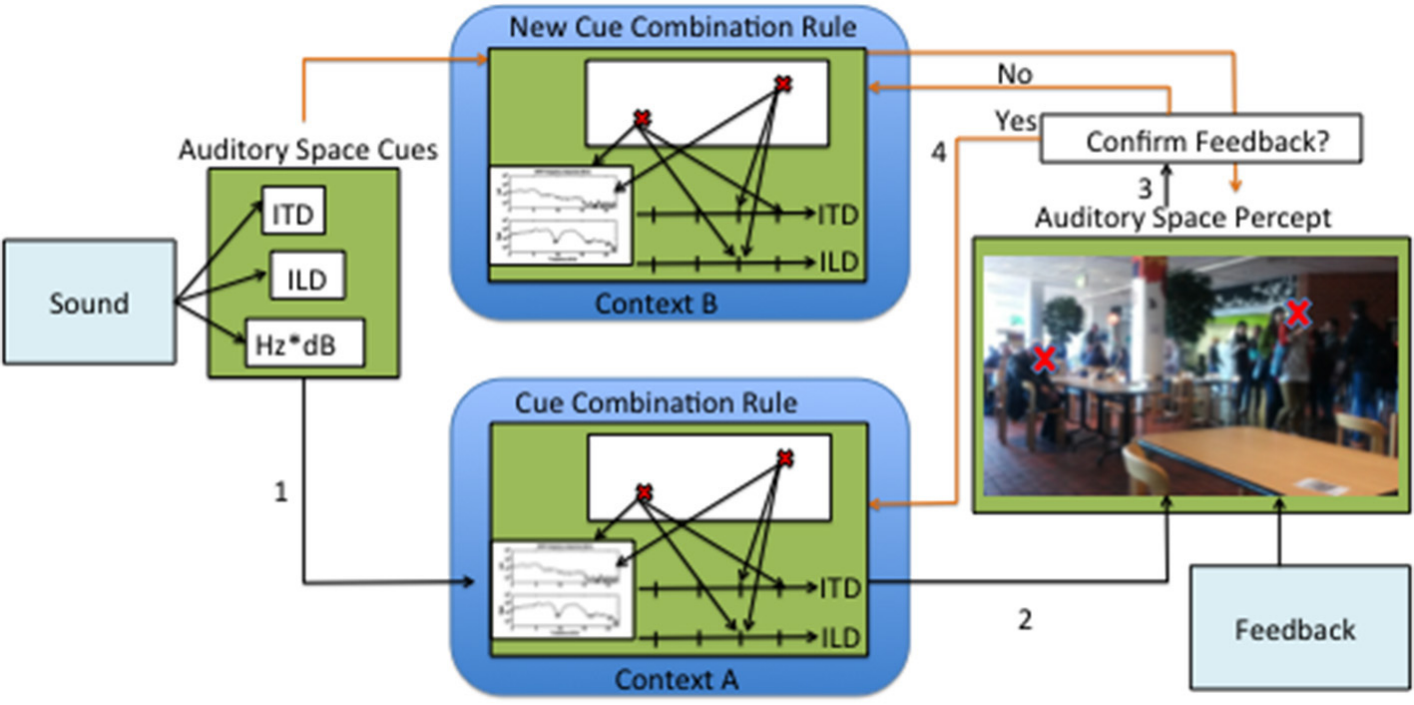
**Illustration of a hypothetical process of auditory adaptation through continuous sensory experience**. First the input sound is decomposed into auditory space cues, then (1) a correspondence is established between the cues and a point in perceptual space. After a correspondence is established (2) a percept is formed. Perceiving auditory sources in space is most often accompanied by feedback. The feedback is compared to the auditory space percept (3). If no differences are found, then there is further tuning of the original cue combination rule. If the feedback is substantially different from the percept, then a new cue combination rule is created.

A hypothetical process of continuous calibration and creation of new cue combination rules is proposed in Figure 1. First, the direction-dependent cues are extracted from the auditory stimulus in the brainstem. These cues then are combined and the source position is estimated, having in mind the contexts and quality of the cues. It is unclear where this process could take place in the brain, but it is likely that the AC plays a special role. Crucial to this process is the continuous feedback, provided by concomitant visual and motor cues. As approached in this review, this feedback can also take the form of a response feedback or direct specification of where the sound should be perceived. The cross-correlation between the auditory source percept and the perceived source position from other senses, namely vision, most likely requires the activity of the SC. However, it may be arguable that in cases of active learning or training with feedback, other cortical areas may also be involved. We propose that with each localization process there is a loop of confirmation or rejection of the cue combination mechanisms. It is precisely from this loop that auditory space adaptation can occur.

## Concluding remarks

Understanding how humans adapt to altered head-related auditory cues is a topic of growing relevance. Firstly, such adaptation processes should be acknowledged. There is a general lack of understanding on how humans deal with hearing loss, hearing surgery, hearing aids, and new hearing technologies. There is accumulated evidence that subjects will adapt to changes in the head-related localization cues. If provided with enough time to adapt, with several days of continuous exposure, subjects will change how they localize. This applies, for instance, to hearing impaired people that preserve their localization abilities, despite interaural sensitivity imbalances (e.g., Keating and King, 2013). On the other hand, assessing new devices or interventions when subjects first experience them may lead to discouraging results, since time is crucial for adaptation. Here we have demonstrated that subjects can also adapt to cue alterations in a short period of time. For that, training programs can be devised to boost the adaptation. Such training programs can use either feedback or active learning, but we found that active learning or combined programs may lead to faster adaptations.

Auditory space learning is an ongoing lifelong process. We proposed that most likely humans are able to represent several auditory cue combination rules at once. This useful skill will allow subjects to adapt to new hearing devices and contexts, and switch between them without experiencing localization disruption. It might ultimately become useful in assistive technologies using augmented reality, where both virtual cues and natural cues are present at the same time. If confirmed, this finding opens perspectives for a future in hearing assistance that accounts for, and integrates, auditory adaptation processes.

### Conflict of interest statement

The author declares that the research was conducted in the absence of any commercial or financial relationships that could be construed as a potential conflict of interest.
